# Metastatic Melanomas Express Inhibitory Low Affinity Fc Gamma Receptor and Escape Humoral Immunity

**DOI:** 10.1155/2010/657406

**Published:** 2010-06-28

**Authors:** Joel F. G. Cohen-Solal, Lydie Cassard, Emilie M. Fournier, Shannon M. Loncar, Wolf Herman Fridman, Catherine Sautès-Fridman

**Affiliations:** ^1^INSERM, U872, Microenvironnement Immunitaire et Tumeurs, Equipe 13, Centre de Recherche des Cordeliers, 75006 Paris, France; ^2^UMRS 872, Universite Pierre et Marie Curie, 75005 Paris, France; ^3^UMRS 872, Universite Paris Descartes, 75006 Paris, France; ^4^The Feinstein Institute for Medical Research, North-Shore Long Island Jewish Health System, Manhassett, NY 11030, USA; ^5^Unite d'Allergologie Moleculaire et Cellulaire, Institut Pasteur, 75724 Paris, France; ^6^Department of Immunology, National Jewish Health, University of Colorado, Denver, CO 80045, USA

## Abstract

Our research, inspired by the pioneering works of Isaac Witz in the 1980s, established that 40% of human metastatic melanomas express ectopically inhibitory Fc gamma receptors (Fc*γ*RIIB), while they are detected on less than 5% of primary cutaneous melanoma and not on melanocytes. We demonstrated that these tumoral Fc*γ*RIIB act as decoy receptors that bind the Fc portion of antimelanoma IgG, which may prevent Fc recognition by the effector cells of the immune system and allow the metastatic melanoma to escape the humoral/natural immune response. The Fc*γ*RIIB is able to inhibit the ADCC (antibody dependent cell cytotoxicity) in vitro. Interestingly, the percentage of melanoma expressing the Fc*γ*RIIB is high (70%) in organs like the liver, which is rich in patrolling NK (natural killer) cells that exercise their antitumoral activity by ADCC. We found that this tumoral Fc*γ*RIIB is fully functional and that its inhibitory potential can be triggered depending on the specificity of the anti-tumor antibody with which it interacts. Together these observations elucidate how metastatic melanomas interact with and potentially evade humoral immunity and provide direction for the improvement of anti-melanoma monoclonal antibody therapy.

## 1. Introduction

The analysis of different types of tumor biopsies by immunohistochemistry for the expression of low affinity receptors for the Fc portion of IgG antibodies (Fc*γ*RII) showed that melanomas are one of the rare non-hematopoietic tumors that express Fc*γ*RIIB. This ectopic expression of Fc*γ*RIIB is restricted to metastatic melanoma and is acquired during the metastasis process. The tumor Fc*γ*RIIB found are fully functional. They bind the Fc portion of IgG and retain the ability to initiate an inhibition of cellular phosphorylation cascades. 

We will present the context of this expression of Fc*γ* receptor. We will review the consequences of the Fc*γ*RIIB expression by metastatic melanoma assayed in mouse models. Finally, we will discuss its implications for human therapeutic approaches.

## 2. Fc*γ*R in the Immune System

### 2.1. Extra- and Intracellular Characteristics of Fc*γ*R

**Figure 1:**
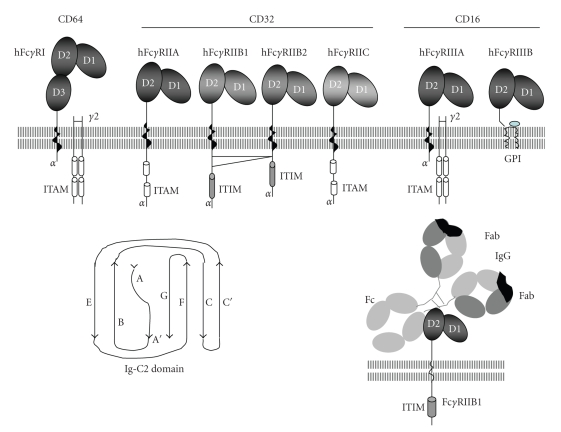
The human Fc gamma Receptors. Structure of Human Fc gamma Receptors CD64, CD32, and CD16. The Fc*γ*R belongs to the Ig superfamily and possess Ig-C2 types of extracellular domains. The domain D2 of the Fc*γ*RIIB binds to the hinge region of the IgG.

The Fc*γ*R are glycoproteins that belong to the Immunoglobulin Superfamily (IgSF) [1]. Their extracellular portions are folded in two or three globular domains called “Ig-C2” domains of approximately 90 amino acids each. A typical “Ig-C2” domain possesses an alternating succession of 8 *β* sheets (A, A′, B, C, C′, E, F and G) and 7 *α* loops (AA′, A′B, BC, CC′, C′E, EF and FG) (Figure 1). The *β* sheets form a bundle stabilized by a disulfide bond between the two opposing B and F *β* sheets. The BC, CC′, EF and FG loops possess asparagines, whose lateral chain NH function are candidates for glycosylation. The Fc*γ*RI possess 3 Ig-C2 domains: the N terminal D1, the D2 and the membrane proximal D3. The Fc*γ*RII and Fc*γ*RIII possess two of these domains: the N terminal D1 and the D2. The D1 is usually the more glycosylated domain [1–3]. Glycosylation represents between 30 and 45 percent of the molecular weight of the Fc*γ*R *α* chain. The Fc*γ*R interact with the immunoglobulin G by their domains D2, which recognize the hinge region of the IgG.

The variations in size and structure of the IgG's hinge region, the nature and degree of glycosylation in the Fc portion of the IgG, and the number of D domains and differences in glycosylation of the Fc*γ*R are parameters that modulate the binding affinity of the different IgG isotypes to each one of the Fc*γ*R (Table 1).

The N-terminal extracellular domain of the Fc*γ*R is followed by a transmembrane domain (TM) of about 20 amino acids and by a C-terminal intracytoplasmic tail called *α* chain, except in Fc*γ*RIIIB. This latter has an extracellular domain covalently linked to a GPI (Glyco-Phosphatidyl Inositol) moiety, which is anchored in the cytoplasmic membrane [4].

The intracytoplasmic domains are more heterogeneous. The Fc*γ*RI, Fc*γ*RIIA, Fc*γ*RIIB2 and Fc*γ*RIIIA are endocytic receptors while the Fc*γ*RIIB1 possess a specific sequence of membrane retention. The Fc*γ*RII are self-sufficient. The intracytoplasmic domain of Fc*γ*RIIA and Fc*γ*RIIC contain a signal-transducing unit called ITAM (Immunoreceptor Tyrosine-based Activation Motif) [5, 6] whereas the Fc*γ*RIIB contain an ITIM (Immunoreceptor Tyrosine-based Inhibition Motif) [7, 8] The Fc*γ*RI and Fc*γ*RIIIA do not possess a signal-transducing unit but form a complex with a dimer of a polypeptide chain containing an ITAM called “FcR *γ* chain” (or Fc*ε*R *γ* chain, as it was first described associated with the FcR for IgE). The association of the *α* chains Fc*γ*RI and Fc*γ*RIIIA with the FcR *γ* chains is required for cell surface expression of these receptors [9] (Figure 1).

### 2.2. Fc*γ*R and Immune Functions

The Fc*γ* Receptors are of high affinity (Fc*γ*RI) when their extracytoplasmic domains recognize monomeric IgG. They are of low affinity (Fc*γ*RII, Fc*γ*RIII) when they loosely recognize monomeric IgG but bind avidly IgG complexed with their antigens (immune complexes) [10–13] (Table 1). All Fc*γ*R belong to the Ig superfamily and share sequence homologies for their extracellular regions, but they differ in their cytoplasmic regions. They are further defined by the way their aggregation initiates or influences the intracellular phosphorylation cascades, which promote cellular activations versus cellular inhibitions. The aggregation of activation receptors (Fc*γ*RI, Fc*γ*RIIA, Fc*γ*RIIC and Fc*γ*RIIIA) initiates the phosphorylation of tyrosine of the ITAM by src-family protein tyrosine kinase (PTK) that recruits kinases like Syk. This leads to the phosphorylation by Syk of downstream targets and to cellular activations, which depend upon the cell type and include cell degranulation, cytokine secretion, and phagocytosis of immune complexes. In addition, the activating Fc*γ*R on monocytes, macrophages, neutrophils, eosinophils, and dendritic cells can mediate killing of IgG-sensitized target cells by ADCC (Antibody Dependant Cell Cytotoxicity). 

Alternative splicing produces two isoforms of the human inhibitory receptors Fc*γ*RIIB. The Fc*γ*RIIB1 possess a 19 amino acid membrane retention sequence absent from the Fc*γ*RIIB2. The Fc*γ*RIIB2 are endocytic receptors expressed by monocytes, macrophages and dendritic cells and are involved in regulation of antigen processing and presentation. The Fc*γ*RIIB1 are non-endocytic receptors expressed in B cells and mastocytes. The inhibition receptor Fc*γ*RIIB1 coaggregation with an activation receptor containing a pITAM, like a triggered BCR (B Cell Receptor), initiates an inhibition message. The pITAM recruits the src-family protein tyrosine kinase Lyn, which phosphorylates the tyrosine of the Fc*γ*RIIB1 ITIM. Then the pITIM recruits the hematopoietic specific inositol phosphatases SHIP, (the ubiquitous SHP1 or SHP2 tyrosine phosphatases can potentially be recruited), which shut down the phosphorylation cascade of activation [14]. Fc*γ*RIIB homoaggregation alone may or may not induce the ITIM tyrosine phosphorylation. In human B cells, it induces the phosphorylation of the ITIM, which leads to cellular inactivation of human peripheral IgM (+) B cells [15]. In mice, it does not phosphorylate the ITIM but induces a Btk dependent apoptosis on class-switched IgG (+) B cells and plasma cells [16–18].

### 2.3. The Fc*γ*R-Dependent Anti-Tumor Defenses

In some patients suffering from cancer lesions, serum IgG antibodies are present that recognize cancer cells, form immune complexes and consequently activate Fc*γ*R. A sustained serological anti-tumor response occurs in patients with melanoma, which includes IgG antibodies against cell surface tumor antigens such as the cancer/testis antigens (NY-ESO-1, MAGE, SSX) and tyrosinase [19–21]. An indication that the serological response is beneficial comes from vaccination studies, which have demonstrated a significant association between the development of an anti-tumor antibody response and survival of melanoma patients [22–24]. The efficacy of therapeutic IgG antibodies against hematopoietic and epithelial tumors argues for an important role of IgG antibodies in anti-tumor defenses [25, 26].

Immune complexes can be involved in the afferent part of the anti-tumor immune response and increase antigen presentation [27, 28]. Moreover, anti-tumor IgG can participate in tumor destruction by activating the cytotoxic activity of Fc*γ*R positive cells. Indeed, in vitro studies have demonstrated Fc-dependant mAb mediated ADCC of cell lines derived from solid and lymphatic tumors by Fc*γ*R expressing monocytes, macrophages, eosinophils, neutrophils, and NK cells [29, 30]. In early studies, the capability of mAb to elicit tumor regression was found to depend on Fc*γ*R-expressing effector cells and the rate of tumor rejection was correlated with the density of Fc*γ*R-expressing effector cell infiltration at the tumor site after antibody therapy [31]. The Ab isotype, which induced the strongest depletion of tumor cells, correlates with the most active subclass in ADCC [30–32]. In a clinical study comparing isotype switch variants of alemtuzumab specific for CD52, the strongest depletion of malignant cells was obtained with the antibody isotype that most effectively induced ADCC in vitro [29]. Studies in Fc receptor deficient nude mice indicate that anti-tumor effects of mAbs, such as anti-CD20 mAb (rituximab) and anti-HER2 (trastuzumab), need the presence of the FcR *γ* chain to be efficient. Macrophages in the lung have been implicated [33]. In the B16 metastatic mouse melanoma model Fc*γ*RI [34] and Fc*γ*RIV(CD16-2) [35], the equivalent of the human Fc*γ*RIIIA, were found to play a major role in the therapeutic effect of the TA99 mAb (a mAb anti-tyrosinase associated protein gp75) [36, 37]. 

The Fc*γ*R-dependent therapeutic effects of IgG anti-tumor antibodies are counterbalanced by the inhibitory Fc*γ*RIIB. Indeed, it was shown that the coexpression of host Fc*γ*RIIB and activation Fc*γ*R on effector cells when engaged together down modulates the anti-tumor efficiency of rituximab and trastuzumab [38].

However, early complement component such as degradation products of C3 might be involved as illustrated by the lower susceptibility of CD11c/CD18 deficient mice to TA99-antibody therapy against mouse metastatic melanoma B16 [39].

## 3. Expression of Tumor Fc*γ*RIIB

### 3.1. Fc*γ*RII and Tumor: First Observations

Since the 1970's, it has been suspected that Fc*γ*R could be expressed by non-hematopoietic cell types and tumors. 

Neural crest derived cells have been reported to express the Fc*γ*R. Examples are the Langehrans cells [40, 41], which express the Fc*γ*RIIB2 [42], the Schwann cells, which express the Fc*γ*RIII [43] and the Purkinje cells, which express the Fc*γ*RIIB [44]. Endothelial and trophoblastic cells of the placenta express FcRn, as well as Fc*γ*RII and Fc*γ*RIII [45, 46]. In Graves' Disease, the thyroid epithelial cells (thyrocytes) express the Fc*γ*RIIB2 [47].

Milgrom et al. first proposed a tumoral expression of Fc*γ*R. They used a cryostat hemadsorption technique to demonstrate that tissue sections of different mouse sarcomas adsorbed antibody-coated sheep erythrocytes (SRBC) [48].

In humans, Tonder and Thunold reported that various non-hematopoietic malignant tumors adsorbed SRBC in a manner dependent on the Fc portion of the antibody [49]. At the same time, Isaac Witz showed the presence of IgG within non-lymphoid tumor tissue and observed that these IgG did not exhibit any antibody activity against tumor antigens, suggesting that they bound to tumor cells via their Fc region [50]. This hypothesis was supported by further experiments where primary cultures of tumors and SRBC formed rosettes [51, 52]. In addition, it was observed that tumor cells injected in mice immunized with non-tumoral antigens were able to bind antibody via their Fc region [53]. However, the ectopic expression of Fc*γ*R by non-hematopoietic tumors cells was controversial because the presence of inflammatory cells, which can express Fc*γ*R, was demonstrated early at the tumor site [54] and because Fc*γ*R expression was lost during short-term tumor cell in vitro culture [55]. Nevertheless, it was shown that the experimental tumors regained expression after a single passage in vivo [56] and that some tumor cell lines express Fc*γ*R [57]. Moreover Gorini et al. described the presence of Fc*γ*R on the cell surface of two human neuroblastoma cell lines using immunohistochemistry and flow cytometry analysis [58]. It is worth noting neuroblastoma are tumors of young children that can arise from any part of the neural crest. 

Using experimental models, it was previously suggested that host factors are involved in the induction of Fc*γ*R expression by tumor cells. Ran et al. observed that anaplastic carcinoma originally induced by polyomavirus (PyV) and BALB/c 3T3 cells transformed in vitro with PyV acquired Fc*γ*R expression only after being passaged in syngeneic animals as solid tumors [56].

### 3.2. Melanoma Express the Fc*γ*RIIb: Immunohistochemical Analysis

The analysis of the expression of Fc*γ*R by non-hematopoietic human tumor cells has been realized by immunohistochemistry on a small number of frozen sections of biopsies [59]. Later on, the technique was adapted for 259 primary tumors and 187 metastatic lesions from 12 different cancer types on paraffin-embedded sections with an anti-Fc*γ*RIIB rabbit antibody specific for the intracytoplasmic domain of the receptor [60]. The morphology of the cells and double staining with a tumor marker allowed identification of the expressing cells as cancer cells and not infiltrating cells. The analyses show that 24% (49/203 biopsies) of melanoma lesions were positive for the expression of Fc*γ*RII. Metastatic melanoma appeared as the tumor with the highest percentage of Fc*γ*RIIB expression at 34% (45/121 biopsies), far more than primary melanoma at 5% (4/82) and other non-hematopoietic carcinoma (ovary, brain and colon carcinoma counted, respectively, at 14%, 5% and 4% positive biopsies). By comparing the origin of the lesions, it appears that liver and lymph node metastases with, respectively, 69% and 44% of positive tumors express higher levels of Fc*γ*RIIB while lung and skin metastasis express lower levels of 20% and 10%, respectively. This global pattern leads to the proposition that the expression of Fc*γ*RIIB correlates with the metastatic progression of the tumor. This hypothesis is reinforced by the finding that patients, for which there are time point biopsy histories, show an absence of Fc*γ*RIIB expression in primary lesions and an expression at metastatic stages only in the liver and lymph nodes, and not in the skin [60].

### 3.3. Gain and Stability of Fc*γ*RIIB Expression by Melanoma Cells

It is still unknown how non-hematopoietic tumors acquire the expression of Fc*γ*RII. Viral induction has been proposed by Witz for anaplastic carcinoma induced by polyomavirus (PyV). In mouse 3T3 cells transformed by PyV, expression was acquired in vivo and lost in vitro [56]. The loss of Fc*γ*RIIB expression following in vitro culture was reported for other non-hematopoietic expressers like Schwann cells or Purkinje cells [61].

When cells derived from frozen biopsies were selectively enriched for the Fc*γ*RIIB1 expression and positive clones were established as cell lines in vitro, the phenotype analysis confirmed the melanoma identity of most of these cell lines by the expression of melanoma marker (GD2, Mel/14) and the absence of hematopoietic cells marker (CD45, CD14, CD1a, CD4, CD15, CD20). The analysis of these cell lines karyotypes, which never showed tetraploidy but presented very specific melanoma chromosomal abnormalities, reinforced the identification and excluded fusion with hematopoietic cells. Interestingly, the expression of Fc*γ*RIIB was stable in culture in vitro for several years. The biochemical analysis established the expression of only the Fc*γ*RIIB1 isoform [59].

Several hypotheses can be proposed to explain the acquisition of Fc*γ*RIIB1 expression at the metastasis stage. One possibility might be that the presence of IgG-containing immune complexes within tumors may upregulate Fc*γ*RII expression on tumor cells, a phenomenon previously observed in vitro in cell lines of hematopoietic origin [62]. It could be that cytokines in the tumor microenvironment may induce the expression of Fc*γ*RIIB1 on tumor cells. One example is IL-4, a cytokine known to upregulate Fc*γ*RIIB on human macrophages [63]. Interestingly, melanocytes are of neural crest origins and melanoma express *de novo* some neural crest cell specific proteins, like bone morphogenic proteins BMP-4 and BMP-7, expression of which is dependent on the master transcription factor Ets-1. BMP proteins are expressed in metastatic melanoma and are prometastatic [64]. It could be that Fc*γ*RIIB are derepressed or actively transcribed by a similar dedifferentiation process.

## 4. Physiological Consequence of Melanoma Fc*γ*RIIB1 Expression

### 4.1. Effect of PyV-Induced Fc*γ*RIIB on Carcinoma Metastasic Behavior

Witz's experiments suggested that the induction of Fc*γ*RIIB in vivo in PyV induced carcinoma would be the result of a positive “selection” by the gain of metastatic or proliferative capabilities, a process called immunoediting of tumors [65]. Zusman et al. demonstrated that in 3T3 cells transfected to express the mouse Fc*γ*RIIB1, the mouse Fc*γ*RIIB2 or a deletant for the intracytoplasmic domain of the mouse Fc*γ*RIIB, only the full Fc*γ*RIIB conferred enhanced tumorigenicity (the IIB1 more than the IIB2) [66] and that the intracellular domain of the receptor was involved in the phenotype [67]. The hypothesis was that the Complement Dependant Cytotoxicity (CDC) and the ADCC defense mechanism would be impaired by the tumor Fc*γ*RIIB1. It was not clear to which extent the intracytoplasmic domain of the receptor was needed. It could be necessary for the receptor retention at the cell membrane, for the signaling through the ITIM and the triggering of phosphatase SHP or for both of these properties of the IC domain.

### 4.2. Xenograft of Fc*γ*RIIB1^+^ Metastatic Melanoma in Immunocompromised Mice

To evaluate the effects of tumor Fc*γ*RIIB1 expression on melanoma growth and metastasis, the tumors derived from biopsies were grafted to immunocompromised nude and SCID mice [59]. Nude mice are impaired for the T cell response but able to mount a B cell xenogenic response characterized by the secretion of antimelanoma antibodies, principally of the mouse IgM and IgG3 isotypes. These mouse immunoglobulins have no affinity for mouse Fc*γ*R but mIgG3 binds human Fc*γ*R. SCID mice are unable to engage in BCR and TCR gene recombination; they show no mature T or B cells, have no serum Ig titer and reject neither allo- nor xenografts. Spontaneous expressers of human tumor Fc*γ*RIIB1 as well as transfectant melanoma cell lines were inhibited in their subcutaneous growth in nude mice. This inhibition was dependent of the intracytoplasmic domain of the human Fc*γ*RIIB1 as transfected melanoma expressing human Fc*γ*RIIB1 lacking the intracytoplasmic domain were not affected in their growth. In SCID mice as well as in vitro, the proliferation of the tumors were unaffected by the Fc*γ*RII expression. Antimelanoma mIgG3 bind to the tumor antigen by its Fab region and to the human tumor Fc*γ*RIIB1 by its Fc region. This crosslink triggered the phosphorylation of the ITIM domain of the receptor, whose pTyrosine interacts with the SH2 domain of SHP-2 (no co-precipitation of SHP-1 or SHIP was detected in HT144 melanoma cell line). The phosphatase activity inhibits cell proliferation and tumor development. This system does not trigger the immune system of the host, as mouse Fc*γ*R have no affinity for mouse IgG3 (Table 1), and ADCC is bypassed. One intriguing question is relative to the role of SHP-2. The effects of upregulation of SHP-2 are controversial; some studies attribute to SHP-2 overexpression an increase in metastatic potential [68, 69], others a decrease [70]. In particular, in a recent study on lung adenocarcinoma, SHP-2 upregulation has been associated with an inhibition of migration (by phosphorylation of Hef1/Cas-L), which suggests that a downregulation of SHP-2 expression could be associated with a gain of migratory potential [70]. One can ask if highly metastatic melanoma, which express Fc*γ*RIIB1, would not be low SHP-2 expressers.

### 4.3. Allogenic and Syngenic Graft of Mouse Fc*γ*RIIB1^+^ Melanoma in Immunocompetant Mice

To evaluate the immunologic host-tumor relationship a murine model was used [60]. The C57BL/6 (H2b) melanoma cell line B16 is indeed slightly immunogenic and as a pigmented cell line, extremely sensible to the effect of anti-tyrosinase antibodies [71]. With this model, a hallmark of success in immunization protocols or passive transfer of anti-tyrosinase antibodies is post-treatment vitiligo [72]. The B16 melanoma transfected to express the mouse Fc*γ*RIIB1 receptors either intact or ITIM invalidated by Y→A mutation were grafted in syngeneic host C57BL/6 mice [60]. The tumor uptake and the growth in the syngeneic host were indifferent to the presence of mFc*γ*RIIB1. In the allogeneic host BALB/c (H2d) (albino mice that do not express tyrosinase [73]), the B16 tumor was strongly rejected and purified anti-B16 antibodies from BALB/c mice were sufficient to transfer the rejection of B16 in SCID mice in an ADCC dependent way [60]. The more striking observation was the ability of B16 (Fc*γ*RIIB1) or B16 (Fc*γ*RIIB1 ITIM^Y→A^) grafted subcutaneously in BALB/c mice to counteract the allogenic rejection and grow (Joel Cohen-Solal, unpublished data). These results propose that Fc*γ*RIIB1 possess the ability to protect the melanoma from the humoral antimelanoma immunity and from the allogenic rejection. This later consideration could highlight the mechanism of allogenic tolerization of the fetus proposed by Clark Anderson. The yolk sac membranes, which are of fetal origin, express FcRn and Fc*γ*RIIB as well as paternal/maternal MHC molecules and are protected from maternal allogenic antibody mediated cell cytotoxicity.

## 5. Mechanism of Action of Melanoma Fc*γ*RIlB

### 5.1. Inhibition of ADCC In Vivo and In Vitro

**Figure 2:**
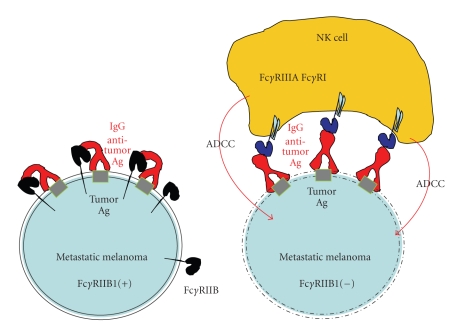
Tumor Fc*γ*RIIB1 expression protects metastatic melanoma from ADCC. Metastatic melanomas that express the Fc*γ*RIIB1 are resistant in vitro and in vivo in immunocompetent mice to the ADCC mediated by FcR *γ* chain dependent effector cells (NK cells, neutrophiles monocytes, or macrophages). The tumor Fc*γ*RIIB1 are decoy receptors. The Fc portions of anti-tumor antibodies are captured by the tumor Fc*γ*RIIB1 and cannot interact with the Fc*γ* of the immune effector cells.

In C57BL/6 mice, the growth of the melanoma B16 is inhibited by treatment of the mice with the mouse IgG2a monoclonal antibody TA99 anti-Tyrosinase Associated Protein TYRP-1/gp75 [72]. In this model, the antibody action is entirely dependent of the FcR *γ* chain of the effector cells of the host. These receptors are the Fc*γ*RI, Fc*γ*RIII and Fc*γ*RIV (CD16-2) and the cells that express them are NK cells, macrophages, monocytes and neutrophils [39, 72, 74–76]. The expression of the tumor Fc*γ*RIIB and Fc*γ*RIIB invalidated ITIM^Y→A^ antagonize the therapeutic effect of TA99. The ADCC inhibition is also observed in vitro with human melanomas expressing Fc*γ*RIIB1 full or deleted of its intracytoplasmic domain [60]. This result suggests that the expression of Fc*γ*RIIB1 has the ability to inhibit the ADCC mechanism independently of the ITIM function. The simplest view is a mechanism of decoy receptor: the anti-tumor antibody binds the tumor by its Fab portion while its Fc portion is caught by the tumor Fc*γ*RIIB1 and cannot be recognized by the Fc*γ*R of the effector cell (Figure 2).

### 5.2. Inhibition of Growth In Vivo and In Vitro

**Figure 3:**
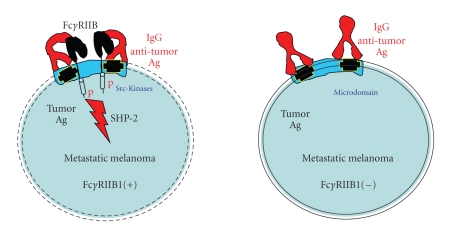
Tumor Fc*γ*RIIB1 expression inhibits metastatic melanoma growth. Metastatic melanomas that express the Fc*γ*RIIB1 receptor are sensible to the action of anti-GD2 mAb. In vitro as well as in vivo in immunocompromised mice the tumor Fc*γ*RIIB1 that are aggregated with the ganglioside GD2 by the mAb are subjected to phosphorylation of the ITIM that recruit the phosphatase SHP-2. SHP-2 dephosphorylates the Protein Tyrosine kinases and initiates an inhibition of tumor growth.

The nevis and primary melanoma express the gangliosides GD3 and GM3. Upon metastasis, they acquire the expression of the enzyme N-acetylgalactosaminyl transferase that converts GD3 into GD2 [77]. Anti-GD2 monoclonal antibodies are used in radiotherapy, coupled to radioelements like ^131^I or ^188^Re. There exist two well-documented mouse anti-GD2 antibodies called 7A4 (mouse IgG3) and 3F8 (mouse IgG3) [78, 79]. Mouse IgG3 bind to human Fc*γ*R and have a cell cytotoxicity, which depends of activation Fc*γ*R and complement receptor of the human host effector cells. They have no effect in vitro on the melanoma proliferation. Surprisingly, the melanomas that express the Fc*γ*RIIB1 are inhibited in their in vitro proliferation as well as in vivo when grafted in SCID mice following treatment with 7A4 [59]. The inhibition is dependent on the intracellular domain of the receptor and the ITIM module is phosphorylated and recruits the tyrosine phosphatase SHP-2 [80]. The simplest explanation of the phosphorylation is the recruitment of a scr-kinase linked to the antigen that is recognized by the anti-tumor antibody, which crosslinks it to the Fc*γ*RIIB1 by its Fc portion. Then the scr-kinase phosphorylates the ITIM, which in the case of the availability of a phosphatase to bind the pITIM, transduces a dephosphorylation wave that inhibits cell proliferation (Figure 3).

### 5.3. The Nature of the Tumor Antigen Matters

These results propose that the Fc*γ*RIIB1 retains its functionality and that its expression could be selected as well as counter-selected depending on the type of antigen recognized by the anti-tumor antibody. Gangliosides like GD3 and GD2 are localized in cholesterol rich microdomains where src-kinases are present. If they attract the Fc*γ*RIIB1 in the microdomains, they may promote the phosphorylation of their ITIM and give a selective advantage to the melanoma that do not express Fc*γ*RIIB1 [59]. Antigens like TYRP-gp75, and Mel14-gp90MEL, which are transmembrane proteins not associated with kinase activities, will not promote the ITIM phosphorylation but rather promote the confinement of the antimelanoma Ab and give a selective advantage to the melanoma that express the Fc*γ*RIIB1 [60].

## 6. Anti-Melanoma Monoclonal Antibodies and Patient Care

### 6.1. Clinical Trials

Several clinical trials are reported in the USA that use antimelanoma mAb; the main targets are GD2, GD3, Mel14 and melanin (http://clinicaltrials.gov/). One protocol is an immunization with an anti-idiotype antibody 4B5 (Ab2), which forms an “internal image” of the antigen GD2. The goal is to mount an anti-GD2 response. The other therapies are based on mAb coupled to radiolabeled elements (^131^I and ^188^Re). With the exception of the anti-idiotype vaccination, the other therapies do not require the immune effector cells of the host, moreover they try to avoid it by the use of F(ab)′2 fragment of the antibodies. These therapies are based on the local irradiation of the cells that are bound to the antimelanoma antibodies. By avoiding the binding to activator Fc*γ*R these antibodies do not deplete the host immune effector cells. For these protocols, the tumoral expression of Fc*γ*RIIB is without effect. For the anti-idiotype vaccination this could partially explain the poor response of patients. In protocols of active vaccination in contrast to induction of anti-tumor IgM, the increase of circulating anti-melanoma IgG was associated with a poor prognosis [81–83].

### 6.2. Optimizing the Fc of mAb

The recent studies of the structure of IgG and their interactions with the different activator Fc*γ*R have allowed the design of optimized antibody Fc fragments, which is already a reality with two products on the market (eculizumab and catumaxomab) and about ten candidates in clinical trial. The main modifications target the glycosylation and the binding sites for C1q and Fc*γ*R. The effects of the modification are evaluated on ADCC, Antibody Dependent Cell Phagocytosis (ADCP) and CDC [84–86]. The treatment of melanoma will benefit from this amazing work realized over ten years, which allows enhanced binding to Fc*γ*RIIA and Fc*γ*RIIIA and lower binding to the Fc*γ*RIIB and which is exactly what is needed to counteract the tumor Fc*γ*RIIB1 expression. An increase of 100-fold in ADCC efficiency is commonly reported with these antibodies [84, 87]. This high level of improvement and the safety of the product will certainly give a second chance to the neglected mAb therapy based on the immune effector function in melanoma treatment.

### 6.3. Modulation of the Immune System with an Anti-Human Fc*γ*RIIB Antibody

The immunization of a mouse transgenic for the human Fc*γ*RIIA with the extra-cellular moiety of the human Fc*γ*RIIB allowed the development of a set of mouse anti-human Fc*γ*RIIB monoclonal antibodies. One of them, 2B6, was selected for its ability to interact solely with the Fc*γ*RIIB without crossreacting with either the Fc*γ*RIIA or the Fc*γ*RIIC and to block the Fc-FcR interaction [88]. 2B6 was modified to reduce its immunogenicity in human by chimerism (xi-mAb) and humanization (zu-mAb). It successfully depletes B cells expressing the Fc*γ*RIIB by ADCC [89]. It can potentially modulate the immune system. It has been postulated that T cell mediated anti-melanoma immunity could be enhanced by B cell depletion as melanoma development is impaired in B cell deficient *μ*MT mice [90]. The use of multi-monoclonal antibody therapy could be envisioned with anti-melanoma antibodies and anti-Fc*γ*RIIB antibodies targeting both melanoma and B cells. However, recent work based on the B16 melanoma model showed the preponderant role of the B cell compartment in the anti-B16 immune response [91]. To address this issue, further studies are needed that will specifically evaluate the extent to which the use of 2B6 (or another anti Fc*γ*RIIB mAb) associated to a monoclonal anti-melanoma therapy could be beneficial.

## 7. Conclusion

The work that we accomplished over the last ten years demonstrated and quantified the ectopic expression of Fc*γ*R by melanoma. We established that Fc*γ*R are selected during the course of the metastasis and that their expression culminates at the later stages in the liver or the lymph nodes. We observed that the expression of Fc*γ*R is restricted to the IIB1 form of receptor, which possess an inhibitor motif ITIM and a membrane retention intracytoplasmic sequence. The expression of the receptor was heterogeneous inside tumor lesions and was dependent on the site of metastasis and most probably to the relation tumor-host. Fc*γ*RIIB1 seem immuno-edited by the presence of activity, which they antagonize in vitro and in vivo in mouse models. In contrast, they are counter-selected in absence of the pressure of effector cells, as observed in vitro and in vivo in nude mice.

On one hand, Fc*γ*RIIB1 are not detected in melanocytes. They are rarely seen in primary tumors where the Fc dependent effector functions are described as impaired. Effectively, in primary melanoma lesion, suppressor T cells and tolerizing dendritic cells (DC) deactivate the immune response. TGF*β*1/2 and IL-10 are the most effective cytokines responsible for the lack of DC maturation [92]. TGF*β* have the ability to downregulate the FcR *γ* chain expression, which reduces the membrane expression of activator Fc*γ*R in myeloid effector cells [93] and IL-10 upregulates inhibitor Fc*γ*RIIB1/2 [94]. Additionally, TGF*β* inhibit IFN*γ* secretion and ADCC in human NK cells [95]. On the other hand, Fc*γ*RIIB1 is highly expressed in metastatic tumors in the spleen or in the lymph nodes [60]. These melanomas have gained a high metastatic power and reside in the liver, which is an environment rich in NK and NKT cells. Tumor Fc*γ*RIIB1 expression appears as a decoy mechanism, which is able to counteract the ADCC mediated humoral immunity. The suppressive effect is powerful enough to allow the allogenic uptake and growth of the C57BL/6 derived B16 tumor in BALB/c mice, which suggest that it should be preponderant in the weak efficiency of the passive transfer of anti-tumor antibodies and of antibody-based vaccination. 

The optimization of Fc domain of therapeutic antibodies is now possible and allows for a higher ADCC, ADCP and CDC potential. In the case of anti-Fc*γ*RIIB1^+^ metastatic melanoma therapy, the optimization will be reached by lowering the Fc binding to the Fc*γ*RIIB binding and by increasing the Fc binding to Fc*γ*RIIIA and Fc*γ*RI. The reality of these efficiency improvements and the innocuousness of humanized antibodies ensure that mAb anti-melanoma approaches will be revisited in a near future.

## Figures and Tables

**Table 1:** Affinity of Fc*γ*R for the IgG. Relative affinity of human and mouse Fc gamma receptors for the different isotypes of human and mouse Immunoglobulins G.

	hFc*γ*RIIB	hFc*γ*RIIA	hFc*γ*RIIIA	hFc*γ*RI
Ka	<10^7^ M	<10^7^ M	1-2 × 10^7^ M	10^8^-10^9^ M
human IgG	3 > 1 = 4 ⋙ 2	HR 3 > 1 ⋙ 2,4	3 > 1 ⋙ 2,4	3 = 1 > 4 ⋙ 2
		LR 3 > 1 = 2 ⋙ 4		
mouse IgG	2a = 2b > 1,3	HR 2a = 2b = 1	3 > 2a > 2b ⋙ 1	2a = 3 ≫ 1,2b
		LR 2a = 2b ⋙ 1		

	mFc*γ*RIIB	mFc*γ*RIII	mFc*γ*RIV	mFc*γ*RI

Ka	<10^7^ M	<10^7^ M	3×10^7^ M	10^8^ M
mouse IgG	1 = 2b > 2a ⋙ 3	2a = 2b = 1 ⋙ 3	2a = 2b ⋙ 1,3	2a ⋙ 1,2b, 3
human IgG	3 > 1 > 2 ≫ 4	1 = 3 > 2 ≫ 4	1,3	3 > 1 > 4 ≫ 2
